# Biosensors Show the Pharmacokinetics of S-Ketamine in the Endoplasmic Reticulum

**DOI:** 10.3389/fncel.2019.00499

**Published:** 2019-11-12

**Authors:** Kallol Bera, Aron Kamajaya, Amol V. Shivange, Anand K. Muthusamy, Aaron L. Nichols, Philip M. Borden, Stephen Grant, Janice Jeon, Elaine Lin, Ishak Bishara, Theodore M. Chin, Bruce N. Cohen, Charlene H. Kim, Elizabeth K. Unger, Lin Tian, Jonathan S. Marvin, Loren L. Looger, Henry A. Lester

**Affiliations:** ^1^Division of Biology and Biological Engineering, California Institute of Technology, Pasadena, CA, United States; ^2^Division of Chemistry and Chemical Engineering, California Institute of Technology, Pasadena, CA, United States; ^3^Janelia Research Campus, Howard Hughes Medical Institute, Ashburn, VA, United States; ^4^Department of Biochemistry and Molecular Medicine, University of California, Davis, Davis, CA, United States

**Keywords:** antidepressants, organelles, green fluorescent protein, protein engineering and design, periplasmic binding proteins (PBPs), inside-out pharmacology, iSketSnFR1, iSketSnFR2

## Abstract

The target for the “rapid” (<24 h) antidepressant effects of S-ketamine is unknown, vitiating programs to rationally develop more effective rapid antidepressants. To describe a drug’s target, one must first understand the compartments entered by the drug, at all levels—the organ, the cell, and the organelle. We have, therefore, developed molecular tools to measure the subcellular, organellar pharmacokinetics of S-ketamine. The tools are genetically encoded intensity-based S-ketamine-sensing fluorescent reporters, iSKetSnFR1 and iSKetSnFR2. In solution, these biosensors respond to S-ketamine with a sensitivity, S-slope = delta(F/F_0_)/(delta[S-ketamine]) of 0.23 and 1.9/μM, respectively. The iSKetSnFR2 construct allows measurements at <0.3 μM S-ketamine. The iSKetSnFR1 and iSKetSnFR2 biosensors display >100-fold selectivity over other ligands tested, including R-ketamine. We targeted each of the sensors to either the plasma membrane (PM) or the endoplasmic reticulum (ER). Measurements on these biosensors expressed in Neuro2a cells and in human dopaminergic neurons differentiated from induced pluripotent stem cells (iPSCs) show that S-ketamine enters the ER within a few seconds after appearing in the external solution near the PM, then leaves as rapidly after S-ketamine is removed from the extracellular solution. In cells, S-slopes for the ER and PM-targeted sensors differ by <2-fold, indicating that the ER [S-ketamine] is less than 2-fold different from the extracellular [S-ketamine]. Organelles represent potential compartments for the engagement of S-ketamine with its antidepressant target, and potential S-ketamine targets include organellar ion channels, receptors, and transporters.

## Introduction

Despite half a century of research and improvement, antidepressant drugs do not work optimally. Although selective serotonin reuptake inhibitor antidepressants help appreciable numbers of patients, their benefits appear too slowly (2–6 weeks) after treatment has begun. In contrast, administration of a single, relatively small (subanesthetic) dose of racemic ketamine for ~1 h partially relieves depression in <1 day; this relief continues for several days post-administration (Berman et al., 2000). In some preclinical studies, R-ketamine has more potent and lasting antidepressant action than S-ketamine (Hashimoto, 2019). Recently, the US FDA approved inhaled S-ketamine for treatment-resistant depression.

However, because higher doses of S-ketamine have adverse effects, developing antidepressants that act similarly to S-ketamine may be a better strategy than using S-ketamine itself. To enable developing better rapidly acting antidepressants, one must first understand the mechanism of S-ketamine action, including the molecular target.

Most investigators emphasize the hypothesis that S-ketamine exerts its antidepressant effects by binding to an N-Methyl-D-aspartate (NMDA) receptor subtype (MacDonald et al., 1991; Blanpied et al., 1997; Preskorn et al., 2008; Autry et al., 2011; Emnett et al., 2013; Gideons et al., 2014; Miller et al., 2014; Johnson et al., 2015). Other articles suggest the following receptor, channel, or transporter targets for ketamine: α3β2 nicotinic receptors (nAChRs; Lee et al., 2012), α4β2 nAChRs (Buisson and Bertrand, 1998), α7 nAChRs (Coates and Flood, 2001; Moaddel et al., 2013), dopamine D2 receptors (Kapur and Seeman, 2001, 2002; Seeman and Kapur, 2003), HCN1 channels (Chen et al., 2009), 5-HT2 receptors (Frohlich and Van Horn, 2014), or 5-HT3 receptors (Yamakura et al., 2000). Most contemporary psychiatric drugs have well-established receptor, channel, or transporter targets. In contrast, the “target” for the antidepressant actions of S-ketamine is poorly understood.

We comment similarly that downstream signaling pathways are poorly understood. Suggested pathways include mechanistic target of rapamycin (mTOR; Zoncu et al., 2011; Moaddel et al., 2013; Miller et al., 2014), eukaryotic elongation factor 2 (EEF2) kinase (Autry et al., 2011; Gideons et al., 2014; Adaikkan et al., 2018), serine/threonine kinase glycogen synthase kinase-3 (GSK-3; Beurel et al., 2011; Liu et al., 2013), calcium/calmodulin-dependent protein kinase II (CaMKII; Adaikkan et al., 2018), brain-derived neurotrophic factor (BDNF; Lepack et al., 2014), Kir4.1-containing transport vesicles (Stenovec et al., 2019), and G-protein translocation to/from lipid rafts (Wray et al., 2018). These molecules are thought to participate in enhancements of glutamatergic (Zanos et al., 2018), cholinergic, or GABAergic (Widman and McMahon, 2018) transmission (Ren et al., 2016). Finally, we comment similarly about brain regions and nuclei. Most studies focus on hippocampus and cortex; but ketamine also blocks bursting in the lateral habenula (Yang et al., 2018).

If one does not know the target for a drug, then an appropriate step is to seek that target in all compartments that contain the drug, and to measure how long the drug remains in each compartment. A previous report shows that a ketamine analog enters cells (Emnett et al., 2016). This report presents the first quantitative, dynamically resolved measurements of S-ketamine in an organelle: the endoplasmic reticulum (ER).

To conduct these experiments, we executed a research strategy resembling our previous report for nicotine (Shivange et al., 2019). We developed a genetically encoded fluorescent biosensor for S-ketamine. We targeted the biosensor to either the plasma membrane (PM) or the ER. We then performed fluorescence measurements to dynamically report the S-ketamine concentration in each compartment.

## Materials and Methods

### Directed Evolution of iSKetSnFR Proteins Using Bacterial-Expressed Protein Assays

Starting with the iNicSnFR biosensor constructs (Shivange et al., 2019), we constructed and measured ~3,000 mutants, in iterative rounds of site-saturated mutagenesis (SSM). We utilized the Quikchange mutagenesis protocol (Agilent), including a mixture of three primers, creating 22 unique codons encoding the 20 canonical amino acids (Kille et al., 2013). The 22-codon procedure yields an estimated >96% residue coverage for a collection of 96 randomly chosen clones.

A Tecan Spark M10 96-well fluorescence plate reader (equipped with appropriate filters) was used to measure resting and S-ketamine-induced fluorescence (F_0_ and ΔF, respectively). Bacterial lysates were tested with excitation at 485 nm and emission at 535 nm. Promising clones were amplified and sequenced. The most sensitive construct in each round of SSM was used as a template for the next round of SSM.

### Measurements on Purified iSketSnFR Constructs

Biosensors selected for further study were purified with the His_6_ sequence included in the bacterial expression vector (Shivange et al., 2019). Proteins were purified by immobilization in phosphate-buffered saline (PBS), pH 7.4, and elution in an imidazole gradient (10–200 mM). Proteins were concentrated by centrifugation through a 30 kDa cut off column, and by dialysis against PBS. The dialyzed protein was quantified using a nanodrop spectrofluorometer, and 50 or (preferably) 100 nM was used in dose-response studies to characterize responses to various ligands. Dose-response relations for ligands were conducted with the plate reader. The pH-dependent dose-response studies with purified iSketSnFR constructs were performed using 3× PBS buffers.

### Expression in Mammalian Cells

We constructed two variants of the iSKetSnFR1 and iSketSnFR2 biosensors for expression in mammalian cells. The plasma membrane (_PM) and endoplasmic reticulum (_ER) variants were constructed by a circular polymerase extension cloning procedure. For iSketSnFR1_PM and iSKetSnFR2_PM, we cloned the bacterial constructs into pCMV(MinDis), a variant of pDisplay (Invitrogen, Carlsbad, CA, USA) lacking the hemagglutinin tag (Marvin et al., 2013). We modified the previous transmembrane domain (Shivange et al., 2019) as follows. We replaced the terminal KKPR of the PDGF receptor (a putative ER retention motif) with KYLQKRRERRRQ (a p14 Golgi export motif) and ENANSFCYENEVAL (a putative Kir2.X ER export motif). To generate iSketSnFR1_ER and iSketSnFR2_ER, we replaced the 14 C-terminal amino acids (QVDEQKLISEEDLN, including the Myc tag) with an ER retention motif, QTAEKDEL (Shivange et al., 2019).

We conducted cDNA transfection experiments on iSketSnFR1_PM, iSKetSnFR2_PM, iSketSnFR1_ER, and iSketSnFR2_ER in Neuro2a cells. Neuro2a cells were purchased from ATCC^1^ and cultured according to ATCC protocols. For chemical transfection, we utilized either Lipofectamine 2000 or Lipofectamine 3000, following the manufacturer’s recommended protocol. Cells were incubated in the transfection medium for 24 h and then in growth media for ~24 h before imaging.

### Expression in Dopaminergic Neurons Differentiated From Human Induced Pluripotent Stem Cells (iPSCs)

Fujifilm CDI^2^ (formerly named Cellular Dynamics International, Madison WI, USA), furnished iCell DopaNeurons. These are human dopaminergic neurons differentiated from induced pluripotent stem cells (iPSCs). The supplier has measured that 89% of the cells are positive for tyrosine hydroxylase by fluorescence-activated cell sorting. The iCell DopaNeurons were maintained in 95% BrainPhys Neuronal medium (STEMCELL Technologies^3^), 2% iCell Neural Supplement B (CDI), 1% iCell Nervous System Supplement (CDI), 0.1% of 1 mg/ml laminin (Sigma), 1% N-2 Supplement 100× (Thermo Fisher Scientific, Waltham, MA, USA) and supplemented with penicillin and streptomycin. iCell DopaNeurons were maintained on dishes for 17–24 days before imaging. Glass bottoms of the 35-mm imaging dishes (MatTek^4^) were coated with ~0.07% poly(ethyleneimine) solution and incubated at 37°C for 1 h. Dishes were rinsed with PBS, then rinsed with water and air-dried overnight. Glass bottoms were then coated with 80 μg/ml laminin solution for 30 min at 37°C before cells were plated. We confirmed that ≥40% of the cells stained for TH by immunocytochemistry using a previously described assay (Srinivasan et al., 2016).

Cultured iCell DopaNeurons were transfected after either 13 or 21 days in culture using the Viafect kit (Promega, Cat. #E4981) at 4:1 transfection reagent (μl): DNA (μg) ratio. The transfection mixture was prepared in 100 μl OptiMEM (Thermo Fisher Scientific, Waltham, MA, USA) containing 4 μl of Viafect transfection reagent and 1 μg of cDNA. The mixture was incubated for 10–15 min, then added directly to fresh maintenance medium in the culture dish. Transfection medium was removed after 24 h and cells incubated for 48–72 h further before imaging.

### Time-Resolved Fluorescence Measurements in Live Mammalian Cells

We find that signals with the iSKetSnFR constructs have brightness similar to those of the previous iNicSnFR cpGFP-based biosensors for nicotine (Shivange et al., 2019), but the dynamic range is somewhat lower for the iKetSnFRs. Datasets were taken on an Olympus IX-81 microscope, in widefield epifluorescence mode. Images were acquired at 3–4 frames/s with a back-illuminated EMCCD camera (iXon DU-897, Andor Technology USA, South Windsor, CT, USA; Pantoja et al., 2009), controlled by Andor IQ2 or IQ3 software. Fluorescence measurements at *λ_ex_* = 470 nm have been described (Shivange et al., 2019). We also installed a second LED for excitation at 405 nm. The epifluorescence cube was previously described (Srinivasan et al., 2011). The 40× lens proved most convenient for imaging several adjacent cells and was relatively insensitive to modest drift of the focus. PM-directed constructs were measured with a region of interest (ROI) that included only the cell periphery.

Solutions were delivered from elevated reservoirs by gravity flow, through solenoid valves (Automate Scientific, Berkeley, CA, USA), then through tubing fed into a manifold, at a rate of 1–2 ml/min. Experiments were performed with HBSS buffer, except that iPSC-derived neurons were studied in PBS plus D-glucose (5.56 mM), MgCl_2_ (0.49 mM), MgSO_4_ (0.4 mM), KCl (5.33 mM), and CaCl_2_ (1.26 mM). Other details have been described (Shivange et al., 2019). As usual in fluorescence imaging experiments, we excluded data from the brightest cells, because these may have fluorescent impurities or aggregates that produce a rapidly bleaching baseline. Data analysis procedures included subtraction of blank (extracellular) areas and corrections for baseline drifts.

### Confocal Fluorescence Imaging

For laser scanning confocal fluorescence imaging, Neuro2a cells were transfected with iSKetSnFR1_PM, iSKetSnFR2_PM, iSketSnFR1_ER, or iSketSnFR2_ER (0.5 μg) with the aid of either Lipofectamine 2000 or Lipofectamine 3000, using the manufacturer’s recommended protocol. The images were acquired with a Zeiss LSM 710 laser-scanning confocal microscope, equipped with a 63× NA 1.4 objective lens. HBSS was used to wash and replace the growth medium in the dishes before imaging. GFP illumination was at 488 nm, observed through a 495–550 nm band-pass filter.

### The S-Slope

We introduce a convenient metric to summarize progress in evolving increasingly sensitive fluorescent biosensors for drugs. The metric, the S-slope, is especially appropriate for low drug concentrations because it corresponds to the relationship between [drug] and ΔF at the beginning of the dose-response relation. We define the S-slope for use with intensity-based drug biosensors:

$$\text{S}-\text{slope = }\Delta \left(\frac{F}{F_{0}}\right)/\left(\Delta [drug]\right).$$

We state the S-slope in units of μM^−1^.

This article uses the S-slope for measurements on S-ketamine biosensors in bacterial lysates, with purified proteins, and expressed in cells. For measurements with bacterial lysates and with purified proteins, it is usually possible to construct a complete dose-response relation with a Hill coefficient close to 1. In this case, we calculated (as in Figure 2A below),

$$\text{S-slope}=\frac{\Delta F_{\max}}{F_{0}}/EC_{50}$$

**Figure 1:**
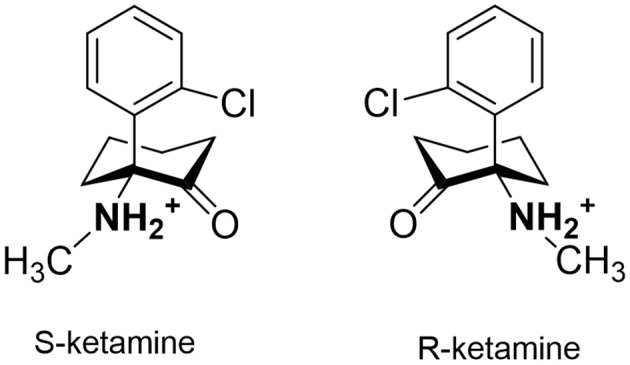
Structures of S-ketamine and R-ketamine.

**Figure 2:**
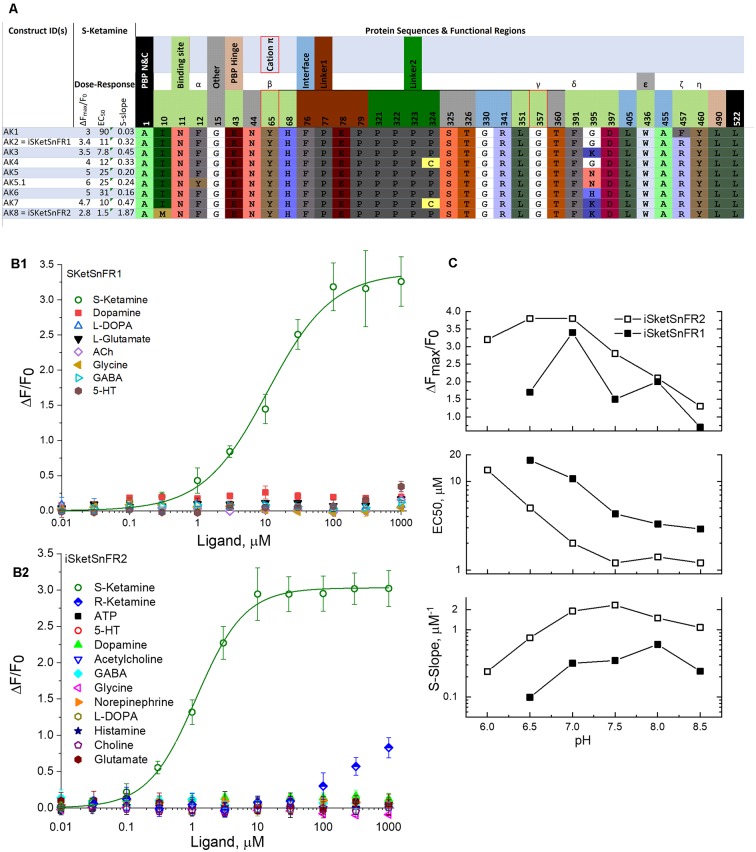
Sequences, dose-response relations, and pH dependence of iSketSnFR1, iSketSnFR2 and related proteins. **(A)** Sequences of eight iKetSnFRs studied. The names iSketSnFR1 and iSketSnFR2 correspond to AK2 and AK8. Functional regions of the biosensor protein are shown as stippled cells above the sequences. Regions highlighted include those surrounding the ligand (“binding site”), the interface between the PBP and the cpGFP moiety, the two linker sequences leading from the PBP to the cpGFP, and vice versa, and the PBP hinge. The N- and C-terminal amino acids are also shown. The numbering corresponds to PDB entry 6EFR (Shivange et al., 2019). The cpGFP moiety, not shown, runs from codon 80 to 320. Greek letters denote aromatic groups that were candidates for cation-π interactions with the N-atom of the ligand (Shivange et al., 2019), and red borders denote those with the strongest evidence. The residues shown were mutated in this study or in a previous study that generated iNicSnFR biosensors. The background colors for amino acids, similar to those in JMOL, have no chemical meaning but are chosen to provide a wide, distinguishing range of colors. There is no correspondence between the background color of the stippled entries and the background color for the codons. **(B1)** Dose-response relations for purified iSketSnFR1, studied for various ligands at pH 7.0, 3× phosphate-buffered saline (PBS; Shivange et al., 2019). The data for S-ketamine have been fitted to the Hill equation, ΔF_max_/F_0_ = 3.4 ± 0.1 and EC_50_ 10.7 ± 1.5 μM, Hill coefficient (n_H_) = 0.91 ± 0.09. The other seven ligands tested yielded responses that were too small for systematic study. **(B2)** Dose-response relations for purified iSketSnFR2, studied forvarious ligands at pH 7.0, 3× PBS (Shivange et al., 2019). The data for S-ketamine have been fitted to the Hill equation, ΔF_max_/F_0_ = 3.0 ± 0.3 and EC_50_ 1.16 ± 0.6 μM, Hill coefficient (n_H_) = 1.18 ± 0.07. The other 12 ligands tested yielded responses that were too small for systematic study. **(C)** Dose-response parameters at varying pH values between 6.0 and 8.5, for S-ketamine at purified iSketSnFR1 and iSketSnFR2. Data are included for curve fits that gave n_H_ values between 0.75 and 1.2 and EC_50_ values < 50 μM. The plots show that iSketSnFR2 has the most favorable S-slope at all pH values studied, because of both its lower EC_50_ and its higher ΔF_max_/F_0_.

### Reagents

All solvents purchased were of analytical grade and used without further purification. S-ketamine HCl was purchased from Sigma-Aldrich (St. Louis, MO, USA; Cat. #K1884, CAS #33643-47-9. We purchased R-ketamine HCl from Cayman Chemicals (Ann Arbor, MI, USA; Cat. #16519, CAS#33795-24-3).

### Data Analysis

Image movie files, spectral data, and dose-response data were analyzed further and presented with general-purpose software. These programs include ImageJ2 (Rueden et al., 2017), Excel (Microsoft), and Origin (OriginLab). All the sequencing analyses used Benchling.

## Results

### Development of iSKetSnFR1 and iKetSnFR2

We tested S-ketamine and R-ketamine (Figure 1) against iNicSnFR1, iNicSnFR2, and iNicSnFR3a, as well as against 12 other biosensors in the series that led to the iNicSnFRs (Shivange et al., 2019). We found no detectable fluorescence increase activated by S-ketamine, at concentrations <100 μM.

For further insights, we computationally docked S-ketamine into the structure of iNicSnFR1 (PDB file 6EFR), and several computationally mutated variants (Supplementary Figure S1). In the highest-ranked results, the predicted distances between the S-ketamine N atom and the aromatic groups are too great to form a cation-π interaction of the type suggested by docking, structural, and mutational studies for the iNicSnFR series with nicotine, acetylcholine, and varenicline (Shivange et al., 2019). These observations, while heuristic and not definitive, suggested that we mutate the aromatic residues.

When we applied SSM to the Tyr357 position, we found S-ketamine responses, but only for a Gly residue at position 357 (ΔF/F_0_ ~0.12 at 1 μM). While insufficiently sensitive for systematic measurements, this construct (AK1) provided an entry for further SSM experiments.

After we identified AK1, further rounds of SSM (retaining the Gly357 codon) led to improvements by mutations at and near the ligand site, including positions 10, 436, and 457. The iSketSnFR1 construct has an S-slope of 0.32 μM^−1^, nearly equal to that of nicotine for iNicSnFR3a and iNicSnFR3b (Shivange et al., 2019). Thus, *in vitro*, one expects a response to 1 μM S-ketamine of ΔF/F_0_ = 0.32. The actual recorded data in cells were in this range (see below). The development series has culminated in iSketSnFR2, which has an S-slope of 1.87 μM^−1^ (Figure 2).

We note the presence of the Phe436Trp mutation (referred to the original OpuBC periplasmic binding protein). One conformer of the Trp side chain can fit into the vacancy left by the absence of a side chain at Gly357. This combination may re-establish a cation-π interaction with the nitrogen of ketamine; further structural analysis would test this hypothesis.

We also note the substantial increased sensitivity for the Met10 codon (from AK7 to iSketSnFR2). We have no explicit structural explanation for the effectiveness of this mutation.

### pH Dependence of iKetSnFRs

Studies of the pH dependence on the GCaMP family provide a mechanistic background for other biosensors that use cpGFP. In the inactive conformation of cpGFP, the fluorophore has a pKa of 8–9, and a second at a higher, only approximately characterized pH. At neutral pH, the fluorophore is almost fully protonated, decreasing the absorption in the band centered at *λ_ex_* ~485 nm (Barnett et al., 2017). In the active form, the pKa is ~7, so that some of the fluorophore molecules are deprotonated. This allows absorption and fluorescence (Barnett et al., 2017). Possibly both the pH dependence of the biosensor and that of the ligand affect measurements with iSketSnFR1 and iSketSnFR2.

Therefore, in the pH range from 6 to 8.5, we determined the ΔF dose-response relations of iSketSnFR1 and iSketSnFR2 using excitation at *λ_ex_* = 485 nm (Figure 2C). The greatest S-slope occurs at pH 7.0–8.5, resulting from maximal ΔF_max_/F_0_ at pH 6.5–7 and an EC_50_ that decreases monotonically with pH. Both those trends resemble results with the iNicSnFR family (Shivange et al., 2019). For measurements at *λ*_ex_ = 400 nm, see Figure 7 below and Supplementary Figure S2.

**Figure 3:**
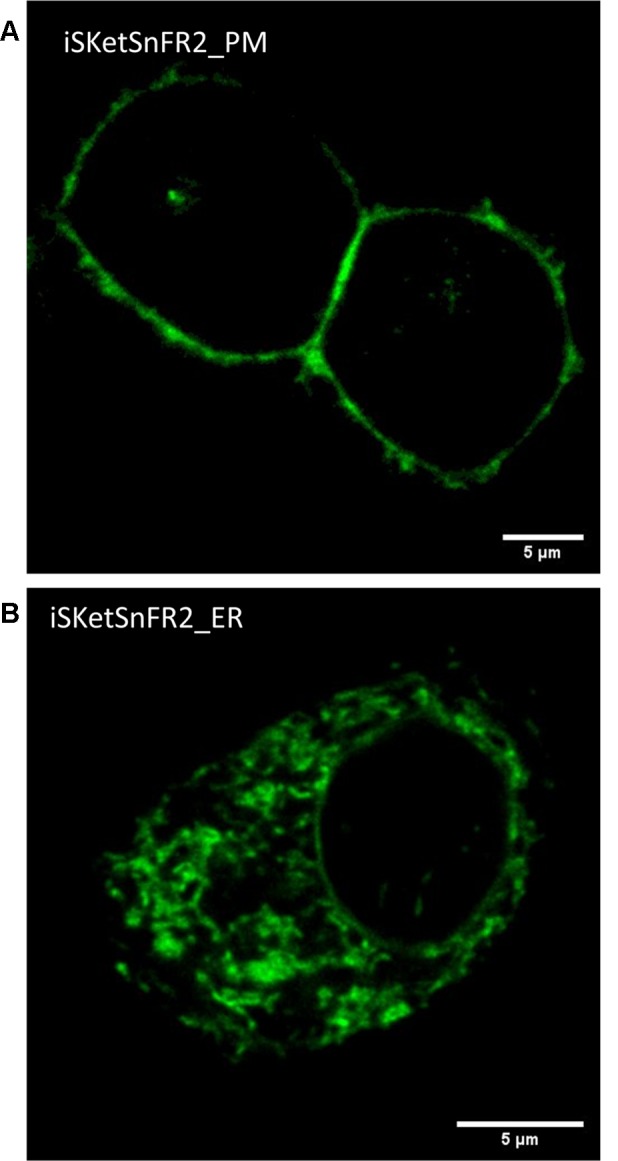
Confocal imaging. **(A)** Typical plasma membrane (PM) fluorescence pattern of a representative Neuro2a cell transfected with iSketSnFR2_PM. Panel **(B)** Typical intracellular fluorescence pattern of a representative Neuro2a cell transfected with iSketSnFR2_ER.

**Figure 4:**
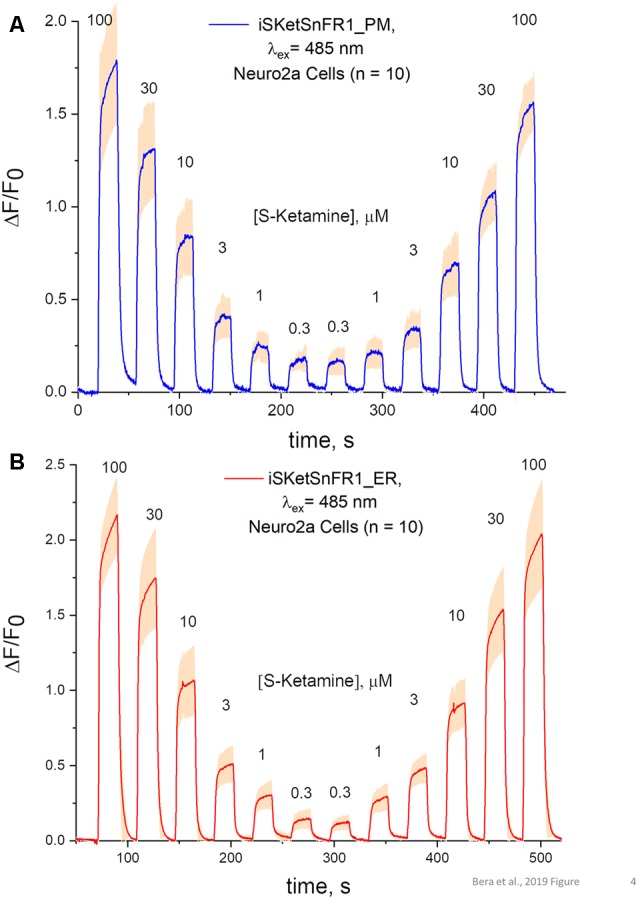
Fluorescence waveforms in Neuro2a cells transfected with iSketSnFR1 constructs. Neuro2a cells transfected with iSketSnFR1_PM or iSketSnFR1_ER were exposed to 20 s pulses of S-ketamine at varying concentrations, at intervals of 40 s. A descending concentration series was followed by an ascending series. **(A)** iSKetSnFR2_PM, average of 10 cells ± SEM. **(B)** iSketSnFR2_ER, average of 10 cells ± SEM.

**Figure 5:**
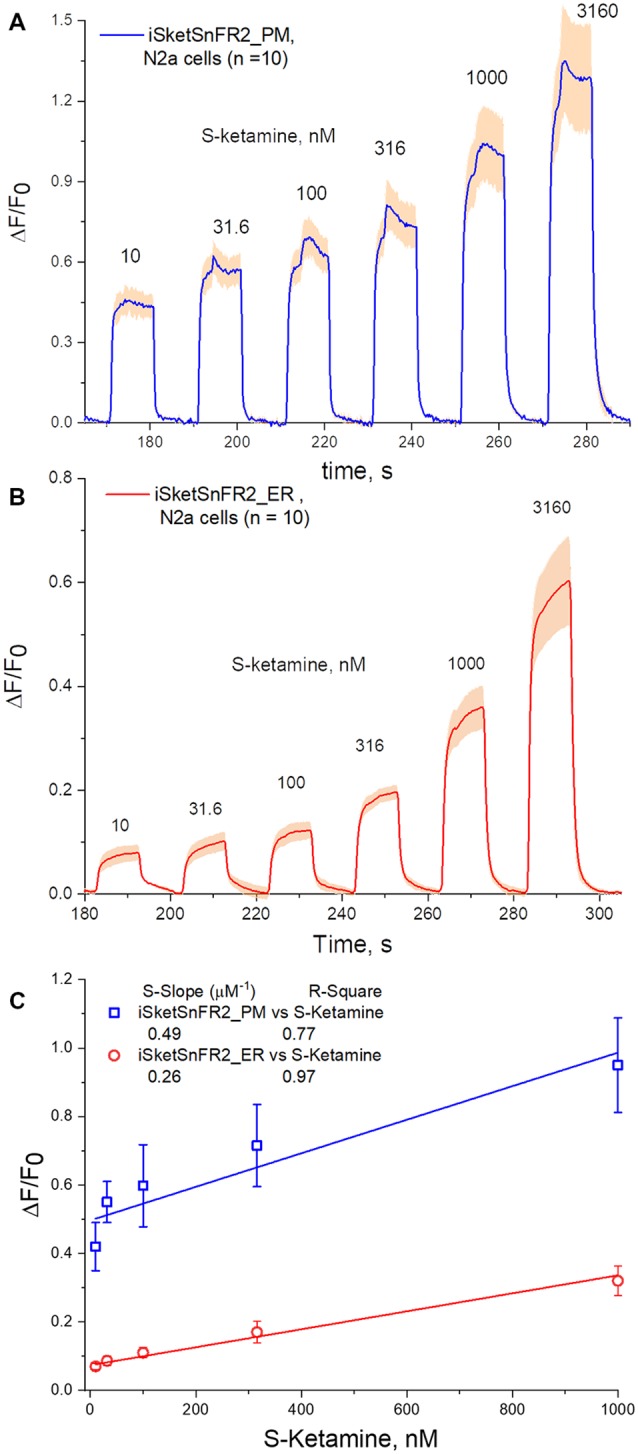
Fluorescence waveforms in Neuro2a cells transfected with iSketSnFR2 constructs and exposed to sub-μM S-ketamine. Transfected Neuro2a Cells were exposed to an ascending concentration series of 10 s pulses of S-ketamine at intervals of 20 s. **(A)** iSKetSnFR2_PM, average of 10 cells ± SEM. **(B)** iSketSnFR2_ER, average of 10 cells ± SEM. **(C)** S-slope calculations from linear fits to the ΔF/F_0_ data for the final 5 s of each application.

**Figure 6:**
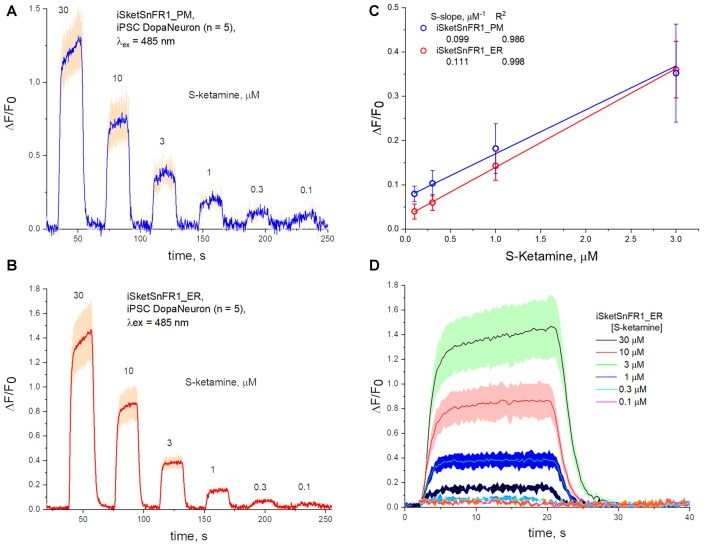
Fluorescence waveforms in induced pluripotent stem cells (iPSCs) transfected with iKetSnFR1 constructs. Dopaminergic neurons differentiated from iPSCs were transfected with **(A)** iSketSnFR1_PM or **(B)** iSketSnFR1_ER. S-ketamine was perfused at varying concentrations for 20 s, at 38 s intervals. Average of five cells, ± SEM. **(C)** S-slope calculations. **(D)** Averaged waveforms for **(B)** on an expanded time axis.

**Figure 7:**
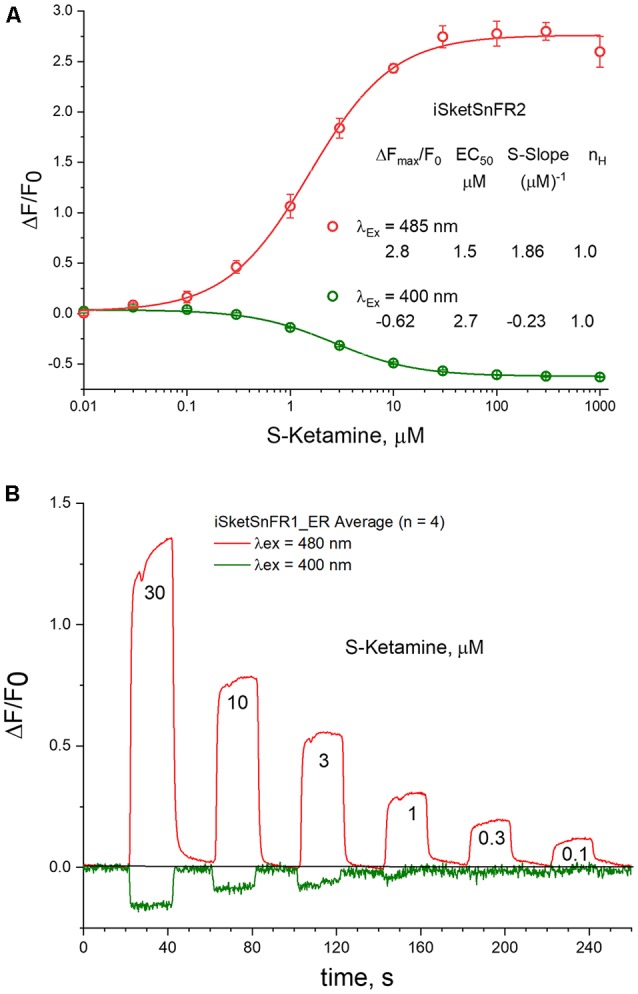
485 vs. 400 nM excitation. **(A)** Dose-response relations in solution for iSketSnFR2, excited at 400 vs. 485 nm. **(B)** Live-cell imaging for iSketSnFR1, with either 485 nm or 400 nm excitation. Pulses of varying S-ketamine concentration lasting 20 s, at 40 s intervals.

The permanently charged nicotine analog, N’-methylnicotinium, previously provided additional insights for the iNicSnFR family (Shivange et al., 2019). The analogous S-ketamine derivative, N,N-dimethyl-S-ketamine, did not produce robust activation of the iSketSnFR constructs, vitiating experiments to study the possible role of charge at the nitrogen atom. This is consistent with but does not prove a reduced role for cation-π interactions between S-ketamine and the biosensor. Regardless of the underlying mechanism, the data suggest that the pH dependence of iSketSnFR1 and of iSketSnFR2 is dominated by that of the cpGFP moiety rather than by that of the weakly basic ligand, S-ketamine.

### The _PM and _ER Constructs Reach the Intended Organelles

We examined the subcellular localization of the iSKetSnFR2_PM and iSketSnFR2_ER constructs, using confocal microscopy (Figure 3). The iSKetSnFR2_PM construct shows the expected localization at the cell periphery (Figure 3A). The iSketSnFR2_ER construct shows the expected intracellular localization, including the nuclear lamina (Figure 3B). Neuro2a cells are not ideal for distinguishing among organelles, and it is possible that some fluorescence arises from localization in both the ER and Golgi. For both the iSKetSnFR2_PM and iSketSnFR2_ER constructs, we noted clear increases in fluorescence when we added 1.5 μM S-ketamine. We described this increase systematically in the specialized, time-resolved, albeit lower-resolution imaging experiments presented below. Similar images were obtained for iSketSnFR1_PM and iSketSnFR1_ER.

### Time-Resolved Responses to S-Ketamine in Live Cells

The S-slope of iSketSnFR1 for S-ketamine roughly equals that of nicotine for iNicSnFR3a and iNicSnFR3b. As expected from this similarity, iSketSnFR1 provided meaningful time-resolved dose-response relations for S-ketamine, at concentrations >1 μM (Figure 4). Transfected Neuro2a cells readily displayed ΔF within a few seconds after the external solution was switched to one containing S-ketamine; and the fluorescence decreased to baseline within a few seconds after the external solution was switched to a ketamine-free solution. The half-maximal concentration of S-ketamine is ~10 μM, near the concentration measured with purified protein.

The rapid antidepressant effects of S-ketamine occur after peak blood plasma concentrations of 0.2–1 μM, and free brain concentration of S-ketamine may be similar (Lester et al., 2012; Janssen Research and Development, 2019). Although the Hill coefficient near unity implies that measurement at [S-ketamine] >1 μM can be linearly extrapolated to provide meaningful insights for lower [S-ketamine], we sought direct measurements at the pharmacologically relevant [S-ketamine]. Our most powerful and sensitive tool for such a study is iSketSnFR2, with its S-slope of 1.9 μM^−1^ at purified protein. Transfected Neuro2a cells readily displayed measurable ΔF within a few seconds after the external solution was switched to an S-ketamine solution; and the fluorescence decreased to baseline within a few seconds after the external solution was switched to a ketamine-free solution (Figure 5). We plotted data for [S-ketamine] ≤1 μM, which is less than the EC_50_ measured with purified iSketSnFR2 protein. This ensures that our measurements remain on the linear part of a conventional dose-response relation. Summarizing our experiments on cells expressing targeted iSketSnFR2 constructs for [S-ketamine] <1 μM, iSketSnFR2_PM displayed an S-slope = 0.42 ± 0.14 μM^−1^ (mean ± SD, 25 total cells from two independent transfections) and iSketSnFR_ER displayed an S-slope = 0.29 ± 0.04 μM^−1^ (mean ± SD, 25 total cells from two independent transfections). These S-slopes do not differ significantly.

### Time-Resolved Responses to S-Ketamine in iPSC-Derived Dopaminergic Neurons

We also studied iPSCs differentiated to become dopaminergic neurons (Shivange et al., 2019) and transfected with either iSketSnFR1_PM or iSketSnFR1_ER. In these cells, responses to S-ketamine appeared and decreased within just a few seconds after jumps in the extracellular S-ketamine concentration (Figure 6), resembling the results in Neuro2a cells. Responses increased linearly with concentration when we applied S-ketamine at concentrations < the EC_50_ (Figure 6). The experimentally determined S-slope for iSketSnFR1_PM was 0.1 μM^−1^, or ~4-fold lower than the value measured for iSKetSnFR2_PM in Neuro2a cells. Importantly, iSketSnFR1_ER constructs in iPSCs displayed an S-slope only slightly greater than that of the iSketSnFR1_PM construct.

S-slopes measured for PM and ER constructs in cells are several fold lower than for purified iSketSnFR proteins, as also observed for iNicSnFR constructs (Shivange et al., 2019). Furthermore, S-slopes measured in cells for iSketSnFR2 constructs are ~2- to 4-fold higher than for iSketSnFR1, rather than 5.8-fold higher as measured for the purified biosensor proteins. Both these differences presumably arise because cellular experiments have appreciable contributions to F_0_ from other fluorescent molecules. Further experiments with various optical arrangements and with various cell types are required. The major conclusion is that, for two cell types and for two iSketSnFR biosensor proteins, the ER S-ketamine concentration follows the extracellular concentration, within a few seconds and within 2-fold.

### Excitation at 400 nm vs. 485 nm

Previous studies indicate that cpGFP-based sensors can also provide information when excited at 400 nm (Barnett et al., 2017). In tests at pH 7, we found that the EC_50_ is not markedly different at 400 and 485 nm, as though measurements are detecting a common binding and conformational change event (Figure 7A). The S-slope at pH 7 is −0.23, some 7-fold lower than at 485 nm (and opposite in sign). With iSketSnFR1_ER, we tested whether one can monitor S-ketamine entry into the ER at *λ_ex_* = 400 nm, even though the lower S-slope produces a lower signal-to-noise ratio. As shown in Figure 7B, this is possible, but only at [S-ketamine] in the higher range of the dose-response relation.

In measurements on purified iSketSnFR2, we compared the pH sensitivity for measurements at *λ_ex_* = 400 nm and at *λ*_ex_ = 485 nm. We confirmed that the EC_50_ for S-ketamine does remain approximately equal when tested at *λ*_ex_ = 400 nm vs. 485 nm, increasing at lower pH (compare Figure 2C vs. Supplementary Figure S2). A similar trend was previously noted for iNicSnFR3a. This trend is opposite to the expectation for a response limited only by the fraction of protonated ligand in the solution (Shivange et al., 2019). Therefore, we restate the previous suggestion that the pH dependence of S-ketamine measurements with iSketSnFR sensors is dominated by the pH sensitivity of the biosensor protein, not of the S-ketamine ligand. Because of this sensitivity, the [S-slope] for *λ*_ex_ = 400 nm becomes quite small at pH < 7, never exceeding 0.3 even for iSketSnFR2 (Supplementary Figure S2).

## Discussion

### S-Ketamine in Organelles

As pointed out in the “Introduction” section, in the absence of well-established information about a drug’s target, one needs to know which compartments a drug enters, how quickly, and at what concentrations. The present study establishes that S-ketamine enters the ER within a few seconds after appearing near cells, then leaves within a few seconds after S-ketamine is removed from the extracellular space. The S-ketamine concentration in the ER is less than 2-fold different from that in the extracellular solution. These conclusions arise from data on two biosensor constructs (iSketSnFR1, iSketSnFR2) and on two cell types (Neuro2a and human dopaminergic neurons differentiated from iPSCs).

A previous report shows that ketamine enters cells (Emnett et al., 2016). The pharmacological role of entry into organelles may differ between nicotine and S-ketamine; the former was studied in a previous article on ER permeation (Shivange et al., 2019). For nicotine, the ER is a major compartment relevant for pharmacological chaperoning and upregulation—the early stages of nicotine dependence (Henderson and Lester, 2015). For S-ketamine, if target engagement occurs in an organelle rather than on the PM, that organelle is still unknown. The sigma-1 receptor, a binding site for both R-ketamine and S-ketamine, occurs in the ER (Su, 2019).

Other organelles should also be considered as possible compartments for target engagement by S-ketamine. In 1974, it was first pointed out that weak bases accumulate, perhaps by factors of 100, in lysosomes and other acidic compartments (de Duve et al., 1974). In one suggestion, the relevant compartment(s) for S-ketamine are acidic vesicles (Lester et al., 2015; Stenovec et al., 2019). Uncertainties about the relevant acidic vesicles imply that the relevant pH is between 4.5 (lysosomes) and 5.5 (synaptic vesicles). Further uncertainties about ketamine permeability in the charged state allow for a wide range of intraluminal [S-ketamine] (Trapp et al., 2008). Therefore, it will be important to study intraluminal S-ketamine concentration directly.

The pharmacokinetic literature points out that lysosomes (pH ~4.5), representing just ~1% of a cell’s volume, would accumulate as much weakly basic drug as the entire cytoplasm (Smith et al., 2012). Antipsychotic drugs, which are also weak bases, accumulate in synaptic vesicles (pH ~5.5), and their release by pre-synaptic action potentials has both pre- and post-synaptic consequences (Trapp et al., 2008; Tischbirek et al., 2012; Tucker et al., 2015; Walters and Levitan, 2019). The present data provide the foundation for modifications of iSKetSnFR1 and iSKetSnFR2 that also function in acidic vesicles.

### Other Candidate Ketamine Analogs and Metabolites

Candidate rapidly acting antidepressants include R-ketamine, as well as metabolites such as (2R, 6R)-hydroxynorketamine (HNK) and (2S, 6S)-HNK. Scopolamine also has rapid antidepressant actions (Wohleb et al., 2016). Biosensors tested in our experiments do respond, though quite weakly, to several of these compounds (Supplementary Figure S3). In previous experiments, the iNicSnFR series was “evolved” from initial biosensors characterized by an S-slope of ~10^−5^ (Shivange et al., 2019). The strategy we describe could conceivably be extended to these ligands.

### Technical Considerations for Drug Biosensors

We comment on developing “iDrugSnFRs,” biosensors for synthetic and endogenous drugs. To some extent, the considerations differ from biosensors for endogenous neurotransmitters. For comparisons among intensity-based biosensors such as PBP-based or G protein-coupled receptor (GPCR)-based constructs, this article emphasizes the S-slope, a single metric that summarizes the beginning of the dose-response relation. The S-slope is simply ΔF_max_/F_0_ divided by the EC_50_. The S-slope has dimensions, μM^−1^. Use of the S-slope has the following advantages.

(1) In our experience with isolated cells and *in vivo* systems, two factors usually render it desirable to follow the time course of relatively low drug concentrations. First, the pharmacological half-maximal dose is often less than the EC_50_ that characterizes the fluorescence. The S-slope describes sensitivity in the appropriate concentration range. Second, a full dose-response relation, in organelles of live cells, can be complicated if higher drug concentrations inhibit transporters, short-circuit proton gradients, or saturate buffers.

(2) An increased S-slope [pedantically, an increased (S-slope)] denotes an increased sensitivity. Interference by other drugs or neurotransmitters (again, at rather low concentrations) can be simply stated as the ratio of the S-slopes. This is an useful comparison if either EC_50_, or ΔF_max_/F_0_, or both vary among ligands. In the case of iSketSnFR1 and iSketSnFR2, all other ligands we measured have ΔF responses so low that an S-slope can be approximately determined only by extrapolation at higher concentrations (Supplementary Figure S3). R-ketamine, another ligand of interest which gives detectable responses at concentrations >100 μM, has an S-slope at least 100-fold lower than S-ketamine at iSketSnFR2.

This article shows that S-slope comparisons between data on purified proteins have some predictive value. However, the S-slopes in cells, between iSketSnFR1 and iSketSnFR2, differed by smaller factors than those measured with purified protein, presumably because in cells, endogenous fluorescent molecules increase the F_0_ values.

(3) Use of a single parameter allows one to estimate the lowest analyte concentration observable, especially if one has characterized the fluorescence measurements in one’s imaging instruments. In isolated cells, which have favorable fluorescence properties, we find that our instruments allow ΔF/F_0_ values of 0.1 to be resolved readily. Therefore, an S-slope of 0.3, 1, 3, or 10 (Shivange et al., 2019) allows measurements as low as ~ 0.3 μM, ~0.1 μM, 10 nM, or 3 nM, respectively.

(4) Use of the S-slope is more general than the previous metric, ΔF/F_0_ at 1 μM ligand (Shivange et al., 2019). As noted above, this generality allows ready extensions to experiments that use only sub-micromolar concentrations of drugs. The S-slope can also be applied to decreases in fluorescence, for instance at 400 nm excitation (Figure 7). Because fluorescence cannot become less than zero, ΔF/F_0_ can never become more negative than −1. However, EC_50_ can become so small that S-slope values become more negative than −1 μM^−1^.

Use of the S-slope does require simplifications that occur with both PBP-based and GPCR-based fluorescent biosensors. These simplifications may not occur with less direct sensors such as those that measure Ca fluxes (Ding et al., 2019) or gene activation (Bick et al., 2017). One simplification appropriate to both PBP-based and GPCR-based biosensors: the Hill slope is near unity, so that responses < the EC_50_ remain linear with the [drug].

Straightforward choices based solely on the S-slope are possible because here, as in Shivange et al. (2019), the binding and conformational changes are rapid enough to eliminate concerns caused by the response of the sensor itself. However, very low K_d_ would depart from this experience. In the simplest view, the equilibrium EC_50_ (K_d_) is a ratio of two (possibly composite) kinetic steps, characterized phenomenologically as K_d_ = k_off_/k_on_. In our experience, k_on_ values for OpuBC-based biosensors are ~10^7^/M/s (Shivange et al., 2019). Therefore, K_d_ values < 10^−7^ M (100 nM) are accompanied by k_off_ <1/s. Such values would produce a “lag” of >1 s between the drug concentration and the fluorescence response.

An important final assumption is that *in vitro* and *in vivo* measurements occur at the same pH. The S-slope does vary with pH, because both its numerator and denominator vary with pH (Figure 2C, Supplementary Figure S2; Barnett et al., 2017; Shivange et al., 2019). We would like to extend the iDrugSnFR measurements to acidic organelles. With PBP-GFP-based iDrugSnFRs, this is not yet possible: the S-slope approaches zero.

### Prospects for Developing Improved Rapidly Acting Antidepressants

Knowing that S-ketamine enters organelles will not in itself develop a new ~24 h antidepressant drug. Nonetheless, such data can help test whether novel mechanisms, such as action on intra-organellar targets and subcellular pharmacokinetics, must be considered in developing such drugs. Researchers may wish to test the subcellular pharmacokinetics, targets, compartment of target engagement, and downstream signaling events of other candidate drugs as rapidly acting antidepressants.

## Data Availability Statement

The datasets generated for this study are available on request to the corresponding author.

## Author Contributions

KB, AK, AS, PB, IB, TC, AM, SG, CK and JM: performed experiments. KB, AK, AS, AM, AN, JJ, EL, BC, JM and HL: analysis. BC, JM, LL and HL: research direction. EU and LT: constructs. AN, BC, KB, EL, LL and HL: manuscript preparation and revision. LL, KB and HL: funding.

## Conflict of Interest

LT is the founder of Seven Biosciences. The remaining authors declare that the research was conducted in the absence of any commercial or financial relationships that could be construed as a potential conflict of interest.

## Footnotes

^1^www.attc.org

^2^fujifilmcdi.com

^3^www.stemcell.com

^4^www.mattek.com
