# Supplementary material for: Biosensors Show the Pharmacokinetics of S-Ketamine in the Endoplasmic Reticulum
Source: Front Cell Neurosci. 2019 Nov 12;13:499. doi: 10.3389/fncel.2019.00499 (PMC6874132; doi:10.3389/fncel.2019.00499)
Supplement: Supplementary file 1 [file Presentation_1.pdf]

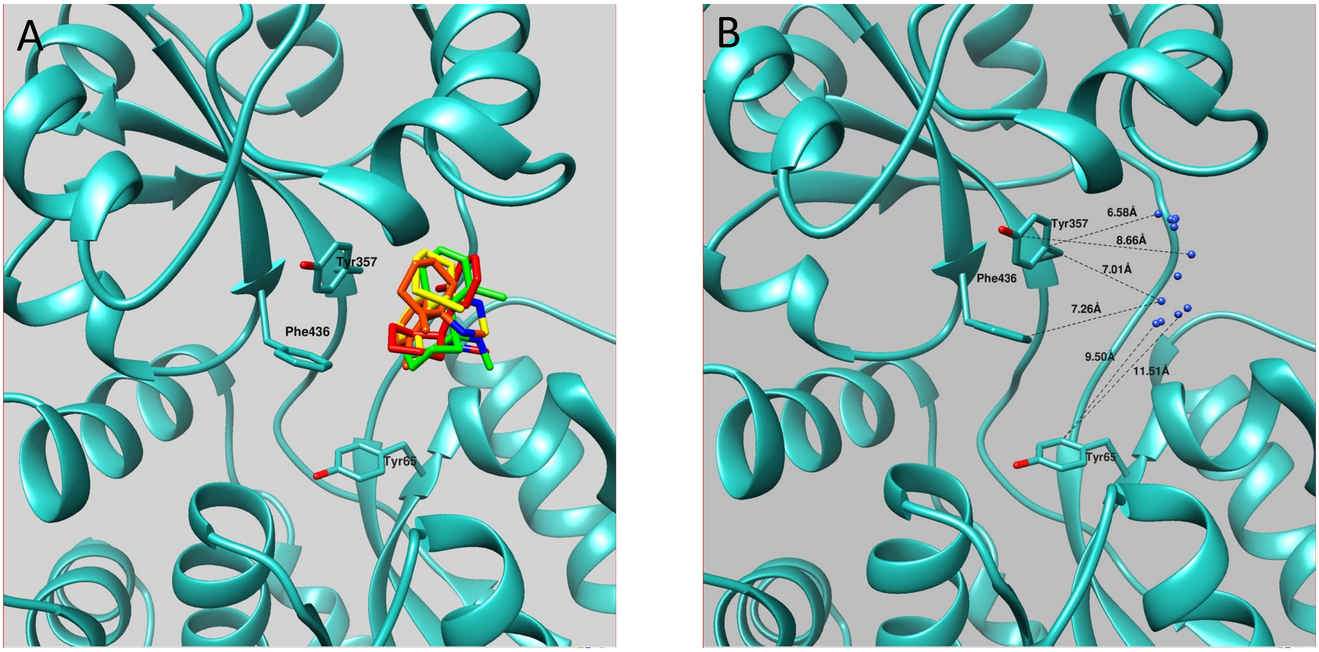

Figure S 1
Docking results for S-ketamine in iNicSnFR constructs
We docked S-ketamine into the structure of an iNicSnFR, iNicSnFR1 (PDB 6EFR) as well as into a computationally mutated protein corresponding to iNicSnFR2 (Shivange et al., 2019)(shown here). Panel A shows three aromatic residues that constituted candidates for the cation-π interaction between nicotine and the binding site in the PBP moiety. These aromatic residues (Tyr65, Tyr357, and Phe436) were termed the α, γ, and ε residues (Shivange et al., 2019). Previous data showed that Tyr357 does interact closely with nicotine (Shivange et al., 2019). However, the docking results suggested that the aromatic moiety of the S-ketamine molecule interacts more strongly with the three aromatic residues shown.

Panel B shows the positions of the nitrogen atoms in S-ketamine for the 12 highest-ranked poses. In all cases, the nitrogen atom was the part of the S-ketamine molecule furthest from the aromatic residues, a result opposite to expectations of a cation-π interaction. The predicted distances between the ketamine N atoms and the aromatic groups were 6.5 to > 9 Angstroms, too distant to form a cation-π interaction of the type that occurs between nicotinic ligands and the iNicSnFR series (Shivange et al., 2019).

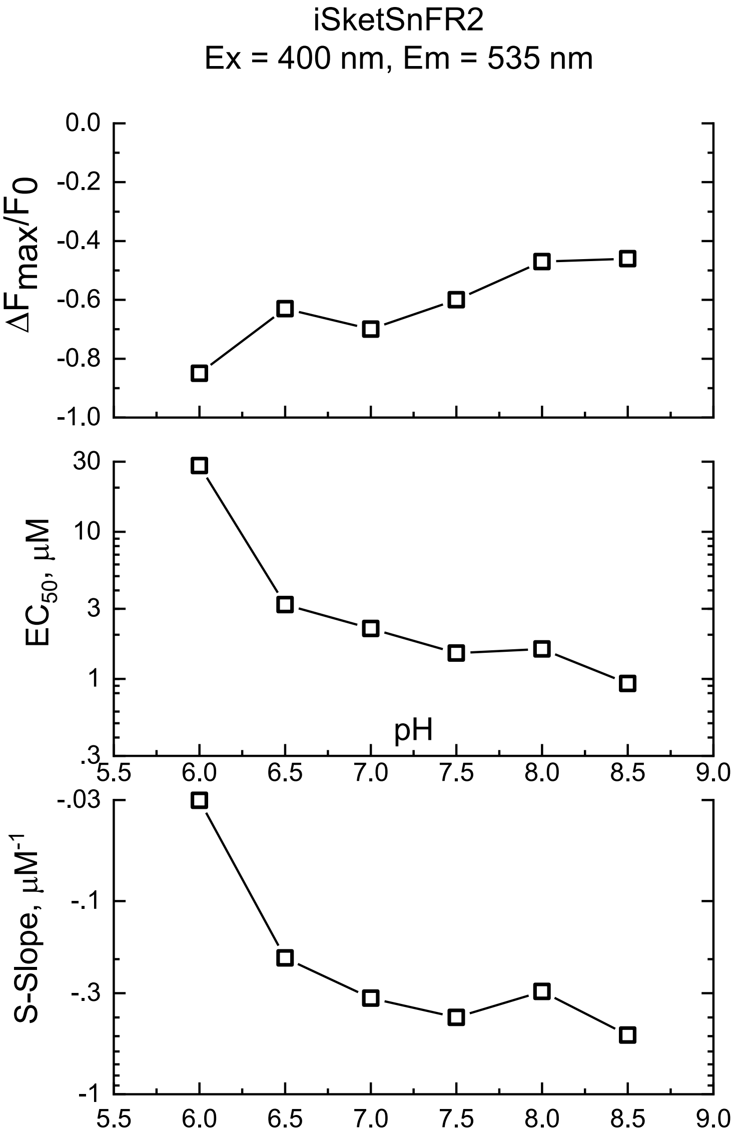

Figure S 2

Dose-response Relations for 400 nm excitation at various pH values
Summary of dose-response relations for iSketSnFR2 in the pH range from 6 to 8.5. Because the $\Delta F/F_0$ values were all relatively small and noisy, we constrained the fits further by assuming a Hill coefficient of 1.0 in all cases. Note that $\Delta F/F_0$ and the S-slope are both negative. Note that EC_{50} and S-slope are plotted on log scales.

**Construct**	scopolamine	(2S, 6S)-HNK	R-ketamine	(2S, 6R)-HNK	radafaxine
AK1	0.01				
AK2			0.005		
AK3					
AK4					
AK5		0.004			
AK6					
AK7					
AK8			0.004		

Figure S 3

Largest S-slopes for rapidly-acting antidepressants other than S-ketamine within the AK1 – AK8 series

S-slopes were extrapolated from measurements on purified proteins, at ligand concentrations between 10 and 100 µM (*e. g.*, data for R-ketamine at AK8, Figure 2B2). AK2 is iSketSnFR1, and AK8 is iSketSnFR2. No members of the series produced detectable responses to (2R, 6R)-HNK or radafaxine. S-slopes have an uncertainty of 2-fold.
